# Ambiguous Genitalia: An Unexpected Diagnosis in a Newborn

**DOI:** 10.7759/cureus.46328

**Published:** 2023-10-01

**Authors:** Ana Losa, Juliana Da Silva Cardoso, Sara Leite, Ana Cristina Barros, Ana Guedes, Cidade Rodrigues, Teresa Borges, Natália Oliva-Teles, Ana Rita Soares, Céu Mota

**Affiliations:** 1 Pediatrics, Centro Materno-Infantil do Norte, Centro Hospitalar Universitário de Santo António, Porto, PRT; 2 Neonatology, Centro Materno-Infantil do Norte, Centro Hospitalar Universitário de Santo António, Porto, PRT; 3 Pediatric Surgery, Centro Materno-Infantil do Norte, Centro Hospitalar Universitário de Santo António, Porto, PRT; 4 Pediatric Endocrinology, Centro Materno-Infantil do Norte, Centro Hospitalar Universitário de Santo António, Porto, PRT; 5 Cytogenetics, Centro de Genética Médica, Centro Hospitalar Universitário de Santo António, Porto, PRT; 6 Genetics, Centro Materno-Infantil do Norte, Centro Hospitalar Universitário de Santo António, Porto, PRT

**Keywords:** ambiguous genitalia, 3p duplication, newborn, disorders of sex development, cri-du-chat syndrome

## Abstract

Alterations in gonad formation or function can lead to congenital conditions in which chromosomal, gonadal, or anatomical sex is atypical. These conditions are referred to as disorders of sex development (DSD) and have a heterogeneous etiology. The assessment of these children by a multidisciplinary team is crucial for an accurate diagnosis and should be initiated promptly due to the potentially life-threatening nature of congenital adrenal hyperplasia, a common cause of DSD. We present a neonate born at 39 weeks with a weak cry, slight hypotonia, poor suction reflex, peculiar facies, and ambiguous genitalia. From the study carried out, the abdominopelvic ultrasound revealed a nodular structure compatible with the left gonad. Aneuploidy screening confirmed the presence of the Y chromosome. Additionally, normal endocrinological studies and the karyotype showed a genotype compatible with cri-du-chat syndrome with partial trisomy of chromosome 3. Children with cri-du-chat syndrome characteristically exhibit a cat-like cry and distinctive facial features, along with developmental delay and intellectual disability. Duplication of 3p is a rare genetic disorder, usually associated with other chromosomal anomalies and congenital malformations, namely, of the genitals.

## Introduction

Disorders of sex development (DSDs) are caused by alterations in gonad formation or function that can lead to congenital conditions in which abnormal chromosomal, gonadal, or anatomical sex is atypical. The incidence of patients with ambiguous genitalia is one per 4,500. The etiology of DSDs is very heterogeneous, and DSDs can be classified as sex chromosome DSDs, 46,XY DSDs, or 46,XX DSDs. 46,XY DSDs include disorders of gonadal development, disorders of androgen synthesis, disorders of androgen action, persistent Mullerian duct syndrome, and some unclassified disorders, such as some complex syndromic disorders. DSD diagnosis requires a careful assessment by a multidisciplinary team (including the specialties of pediatrics, endocrinology, genetics, surgery, and pedopsychiatry) in an experienced center. Evaluation of these children should be undertaken as soon as possible because congenital adrenal hyperplasia, the most common cause of DSDs, can be life-threatening. Furthermore, DSDs are perceived as distressing by most families and often demand psychosocial support [1-2].

## Case presentation

We report a case of a neonate born at 39 1/7 weeks via vaginal delivery to a 28-year-old, healthy, Caucasian primigravida. Early on in the pregnancy, there were no complications, but by the time of 21 weeks of gestation, the cavum septum pellucidum and the corpus callosum were difficult to visualize. Ultrasonography evaluation at 23 weeks of gestation was anatomically normal, and genitalia were presumed to be female. Intrauterine growth restriction (8.6th percentile), with normal umbilical artery Doppler flow, was identified at 35 weeks of gestation. The parents were non-consanguineous, and there was no relevant family history, including genetic diseases. The neonate was born with Apgar scores of 8 and 9 at one and five minutes, respectively, and a birthweight of 2,450 g (7th percentile), length of 46.5 cm (17th percentile), and head circumference of 33 cm (13th percentile).

**Figure 1:**
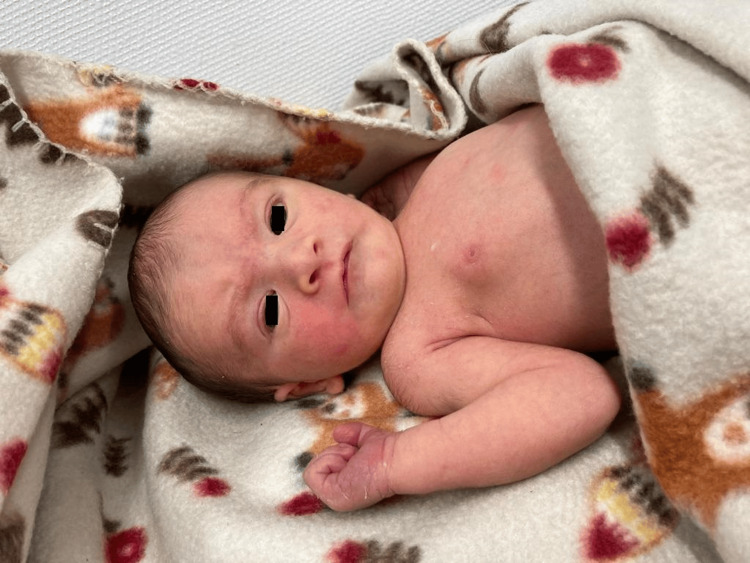
Peculiar facies with a round moon face, broad nasal bridge, hypertelorism appearance, epicanthal folds, low-set ears, and micrognathia.

**Figure 2:**
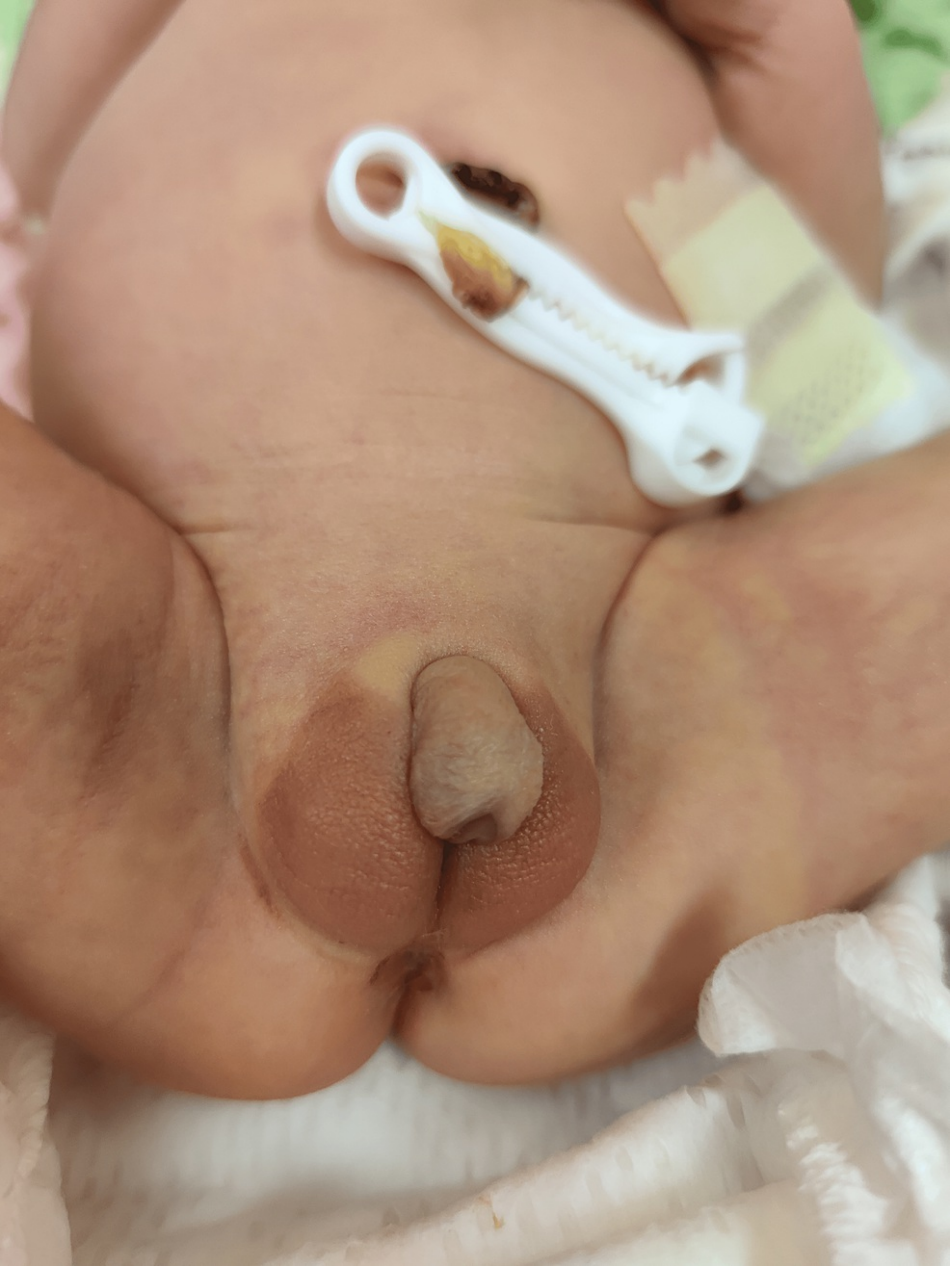
Ambiguous genitalia: phallic structure with fusion of large lips vs underdeveloped scrotal bags.

**Figure 3:**
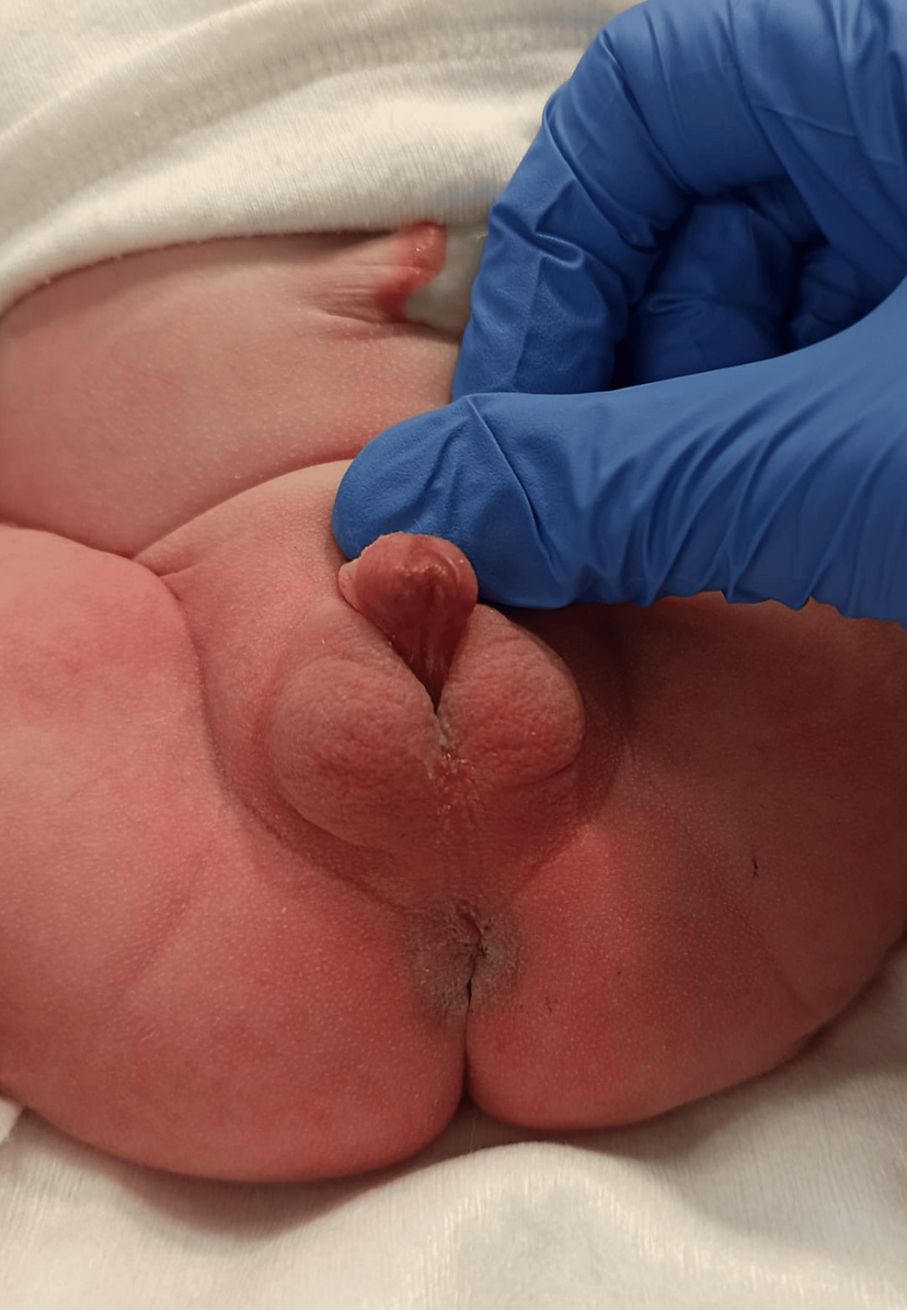
Ambiguous genitalia: urethral meatus in the most proximal region of the phallic structure.

On birth examination, the neonate presented with a weak cry, slight hypotonia, poor suction reflex, and peculiar facies (Figure 1), and genitourinary observation showed ambiguous genitalia: phallic structure with fusion of large lips vs underdeveloped scrotal bags, perineum hypospadias (ureteral meatus in the most proximal region of the phallic structure), gonad palpable in the right inguinal canal, and patent anus (Figures 2-3).

Initial investigation showed venous blood gas with pH 7.38, partial pressure of carbon dioxide of 48.8 mmHg, bicarbonate of 25.3 mmol/L, sodium of 140 mmol/L, chloride of 103 mmol/L, and potassium of 4.3 mmol/L. Abdominal, pelvic, and renovesical ultrasound presented nodular image about 8 x 5 mm adjacent to the right side of the bladder and the right internal inguinal ring, mobilizable to the right inguinal canal, suggestive of corresponding to the gonads; it was not possible to identify a safe image compatible with gonads on the left in echographically accessible plans; no structures compatible with the uterus or ovaries were identified in the evaluation of the pelvis.

The initial cytogenetic analysis confirmed the presence of the Y chromosome with aneuploidy testing by region-specific assay. Cranial ultrasound was normal. The endocrinological study confirmed normal levels of 17-hydroxyprogesterone, follicle-stimulating hormone, luteinizing hormone, antimullerian hormone, testosterone, and dihydrotestosterone.

The result of karyotype with high-resolution banding, followed by multiplex ligation-dependent probe amplification (MLPA) analysis, was 46,XY,der(5)t(3;5)(p26.2;p15.2)mat. This investigation revealed a partial deletion of the short arm of chromosome 5, apparently from segment 5p15.2->5pter, representing a genotype compatible with cri-du-chat syndrome and concomitantly a partial terminal duplication of the short arm of chromosome 3, of the 3p26.2->3pter segment, which represents a trisomy for this region. The father has a normal karyotype. However, the mother is a healthy carrier of a balanced translocation t(3;5); thus, her son has inherited an unbalanced constitution due to chromosomal missegregation.

## Discussion

Cri-du-chat syndrome is a rare genetic disorder first described in 1963, in which a variable portion of the short arm of chromosome 5 is missing or deleted. The prevalence is one per 50,000 live births, and in approximately 80% of the cases, the deletions are de novo [3].

Phenotypically, the affected children have a cat-like cry during the first years of life (providing the name of the syndrome), which is less pronounced with growth. They can also present with low birth weight, hypotonia, and microcephaly. Distinctive facial features may include an abnormally round or moon face, a broad nasal bridge, hypertelorism, strabismus, short and upslanting palpebral fissures, epicanthal folds, low-set ears, and micrognathia. Hypotonia and a weak suction reflex can lead to feeding difficulties. In addition, developmental delay and moderate to severe intellectual disability can be seen [4,5]. The follow-up and treatment rely on a multidisciplinary team, with individualized interventions for each individual [6].

Duplication of 3p is a rare genetic disorder first described in 1972, consisting of an extrachromosomal segment in one of the short arms of chromosome 3. More than 80 people have been described, but in most cases, they had an additional chromosomal abnormality, which makes it difficult to determine which signs and/or symptoms are exclusively related to this duplication. The features described in the literature include microcephaly-like facial features, hypertelorism, a prominent philtrum, and microretrognathia; mild-to-moderate developmental delay; skeletal, heart, and respiratory issues; and genitalia anomalies, mainly in boys, such as micropenis, cryptorchidism, and hypospadias [7].

To our knowledge, there are no reports in the literature of the described genotype with an unbalanced translocation of t(3;5) nor of cri-du-chat syndrome associated with ambiguous genitalia. On the other hand, regarding 3p duplication, there are reports of minor genital anomalies. Thus, in this case, the presented ambiguous genitalia might be related to this rare genetic condition.

## Conclusions

The diagnosis of DSDs must be made early. Aneuploidy screening is essential as a first step toward gender identification. The subsequent study includes endocrinological and genetic studies. Children affected with cri-du-chat syndrome characteristically have a cat-like cry and distinctive facial features with developmental delay and moderate-to-severe intellectual disability. 3p duplication is a rare genetic disorder, usually associated with other chromosomal anomalies, with a phenotype of difficult characterization, craniofacial dysmorphia, and congenital malformations, namely, of the genitals. Patient and family approaches must be carried out by a multidisciplinary team in an experienced center to provide counseling and genetic prenatal diagnosis for an upcoming pregnancy.
